# Altered Sensory Insular Connectivity in Chronic Postsurgical Pain Patients

**DOI:** 10.3389/fnhum.2018.00483

**Published:** 2018-12-05

**Authors:** Yin Ying Ching, Chenhao Wang, Terence Tay, Yng Miin Loke, Phua Hwee Tang, Ban Leong Sng, Juan Zhou

**Affiliations:** ^1^Center for Cognitive Neuroscience, Neuroscience and Behavioral Disorder Program, Duke-NUS Medical School, National University of Singapore, Singapore, Singapore; ^2^KK Women’s and Children’s Hospital, Singapore, Singapore; ^3^Clinical Imaging Research Centre, The Agency for Science, Technology and Research and National University of Singapore, Singapore, Singapore

**Keywords:** pain, functional connectivity, insula, chronic surgical pain, fMRI, acute heat stimulation

## Abstract

Chronic postsurgical pain (CPSP) occurs in up to 50% of individuals after surgeries and 32% after hysterectomy, leading to major adverse effects on quality of life and socioeconomic burden. Little is known about whether and how large-scale neural networks being affected in CPSP, particularly with regard to the functional connectivity (FC) of insula which is known to be the hub of the intrinsic neural network playing a critical role in pain processing. Here, we sought to examine the dynamics of insular FC in the context of noxious stimuli in CPSP patients. To this aim, resting state fMRI data were acquired, before and after acute heat pain stimulation, from 11 individuals with chronic post-hysterectomy pain (CPHP) and 22 age-matched healthy controls (HCs) who had a hysterectomy but without chronic post-surgical pain. We examined whole-brain FC were mapped by seeding at the sensorimotor and chemosensory subfields of the insula and found significant group × stimulation interaction effects. Specifically, the HC group had increased FC between the left sensorimotor insula and right angular and middle occipital gyrus (MOG) and increased FC between the left chemosensory insula and bilateral angular and MOG following pain stimulation. In contrast, such pain stimulation related FC changes were absent in the CPHP group. Furthermore, higher insular FC at baseline and smaller increased insular FC after pain stimulation correlated with clinical pain scores in CPHP patients. Our findings suggest that CPSP is associated with altered dynamics of large-scale functional networks anchored in the insula.

## Introduction

Chronic postsurgical pain (CPSP) is a potential adverse outcome after surgery. It has been described in patients after common surgical procedures and with an estimated incidence as high as 32% after hysterectomy (Perkins and Kehlet, 2000; Kehlet et al., 2006; Brandsborg et al., 2007; Fletcher et al., 2015; Pokkinen et al., 2015; Beyaz et al., 2016; Weibel et al., 2016). The development of chronic pain is thought to be multifactorial. In addition to peripheral persistent noxious stimuli (Li et al., 2007), the transition from acute to persistent postsurgical pain also involves maladaptive neuroplastic changes in neurological pathways (Zeilhofer et al., 2012) and neural remodeling in brain connectivity (Chapman and Vierck, 2017). It has been postulated that persistent aberrant nociceptive activity causes neuroplastic changes in central nervous system networks that subserve pain and attention/cognition functions, ultimately leading to a chronic pain state (Davis and Moayedi, 2013).

Recently, resting state functional magnetic resonance imaging (fMRI) has become the primary tool to understand functional organizations in normal brain and altered large-scale functional networks in brain disorders (Damoiseaux et al., 2012; Wang et al., 2018). From the resting state fMRI data, functional connectivity (FC) has been used to characterize the neural associations between brain regions. FC is defined as the spontaneous synchronization of low-frequency BOLD fluctuations across distributed cortical regions at the resting state (Biswal et al., 1995). Accordingly, the spatial patterns of functionally connected brain regions are referred as intrinsic connectivity networks (ICNs; Fox et al., 2005; Seeley et al., 2007). The salience network (SN) is one of the major ICNs which plays a critical role in emotion and interoceptive autonomic processing (Menon and Uddin, 2010; Chong et al., 2017). Anatomically, the frontoinsular cortex is the key hubs of the SN (Seeley et al., 2007). Altered insular FC to other cortical regions has been reported in chronic pain conditions (Napadow et al., 2010; Baliki et al., 2011; Ichesco et al., 2016). For example, patients with the temporomandibular disorder were found to have increased FC between left anterior insular cortex and pregenual anterior cingulate cortex (Ichesco et al., 2012). Patients with fibromyalgia were also reported to have increased FC in the default mode network (DMN) and SN (Napadow et al., 2010). However, little is known about how the large-scale functional networks such as the SN are affected in CPSP, particularly in the insular sub-regions that perform functional rule in the modulation of noxious stimuli (Kurth et al., 2010).

To cover these gaps, we aim to examine the insular FC changes at resting-state before and after heat pain stimulation in chronic post-hysterectomy pain (CPHP) patients and controls. Here, two regions of insula were selected as the seeds to define the SN: sensorimotor and chemosensory regions. The sensorimotor part of insula has well-established linkage to somatosensory, motor and temporal cortices and is involved in sensory processing including pain (Kurth et al., 2010; Cauda et al., 2011). Chemosensory insula shows activation under chemical stimulus such as olfactory and gustatory tasks and interacts with emotion and memory process (Poellinger et al., 2001; Kurth et al., 2010). We hypothesized that the FC to these two insula sub-regions at resting-state would be altered in CPHP individuals at baseline and after noxious stimuli. We also sought to test the associations between functional abnormalities and clinical severity of pain symptoms.

## Materials and Methods

### Participants

We recruited 11 CPHPs and 22 age-matched healthy controls (HCs) between October 2013 and September 2015. All participants completed pre-operative pain catastrophizing scale questionnaire (Sullivan et al., 1995) and temporal summation testing before undergoing an abdominal or laparoscopic hysterectomy at KK Women’s and Children’s Hospital (KKH), Singapore. They fulfilled the following set of inclusion criteria: (a) 21–70 years old; (b) American Society of Anesthesiologists status ASA 1 and 2; (c) benign conditions such as fibroids or adenomyosis; and (d) Abdominal or laparoscopic hysterectomy. Exclusion criteria include: (a) vaginal hysterectomy; (b) uterine prolapse, endometriosis, malignant disease, the main indication being pelvic pain; (c) failure to determine tonic heat stimulation; and (d) history of drug dependence or recreational drug use. The study protocol for this investigation was approved by the Singhealth CIRB committee (approval number: 2013/512/D). Written informed consent was obtained from all participants who were at least 21 years after a full description of the study was provided.

Four-month postoperative follow up calls were arranged for all participants to assess the presence of CPHP condition and clinical pain scores. Pain score scale is a well-known assessment tool for post-surgical pain (Breivik et al., 2008). It is based on 0–10 ranking scales, with score 0 represents no pain and score 10 represents worst pain imaginable. Participants were assigned to CPHP group if they experience clinical pain scores ≥3 and pain duration >3 months. A cut-off pain score 3 was chosen because pain score > = 3 usually corresponds to at least mild functional interference with patients’ daily activities (Boonstra et al., 2014, 2016). Each participant underwent MRI studies 1–2 months after the follow-up calls.

### Image Acquisition

Participants were asked to cease pain medications other than paracetamol and nonsteroidal anti-inflammatory agents for 48 h prior to MRI. The participants then underwent neuroimaging sessions both before and after tonic heat stimulation, on a 3-T Siemens Skyra system (Siemens, Germany) using a phased-array head coil. The session included the acquisition of high-resolution T1-weighted images and gradient-echo EPI of resting state fMRI pre and post heat stimulation (TR/TE = 2000/35 ms, voxel size = 1.0 × 1.0 × 1.0 mm^3^, 200 repetitions) during which participants were instructed to remain still and keep their eyes on the fixation. Subjects were monitored throughout their time in the scanner for any potential adverse event.

Tonic heat stimulation was conducted by using an FMRI compatible Medoc Pathway Thermosensory Analyzer system (Medoc, Ramat-Yishai, Israel) with a Peltier surface stimulator applied to the left lower thigh. A 44°C stimulation was chosen as the most appropriate one in terms of tolerance of participants during scanning. There were a 44 s break between the first resting state and pain stimulation and another interval of 50 s between the end of the pain stimulation and the second resting state scan. After a warning signal, the temperature rose (5°C/s) to 44°C and stayed at this level for 7 min. The participant then continuously rated the pain score every 30 s (0 = no pain, 10 being the worst pain imaginable) using continuous visual analog scale (CoVAS; Hawker et al., 2011). In the case that any subject found the heat pain intolerable, the test was stopped, and missing points were scored as 10 out 10 on the numerical rating scale. Total scan time was around 1 h, inclusive of 47 mins actual scan time and time for set up of pain apparatus. Participants were monitored throughout their time in the scanner for any potential adverse event. In this study, all participants tolerated the 44°C heat stimulation well without any premature termination of scan.

### Imaging Pre-processing and FC Analysis

The resting state fMRI data were preprocessed using procedures outlined in a previous study (Wang et al., 2018) using the FMRIB Software Library (Jenkinson et al., 2012). The resting-state fMRI data before and after heat stimulation were motion corrected, co-registered to structural MRI, spatially normalized and smoothed using the standard protocol following our previous studies (Zhou et al., 2010). All 11 CPHP patients and 21 of 22 controls passed both the T1 and fMRI head motion quality control measures (i.e., absolute maximum displacement <2 mm).

To investigate the involvement of insular connectivity in CPHP, we adopted a seed-based FC approach (Wang et al., 2016). Kurth et al. (2010) has suggested anatomical and functional differentiation of insula. Based on this independent meta-analysis of task-based fMRI studies of insular function, we estimated whole-brain voxel-wise FC to the sensorimotor and chemosensory seeds covering the mid-posterior and central regions of insula following the approach described in our previous work (Wang et al., 2016). Briefly, the average time series from each ROI was extracted and correlated with the time series of every other voxels by computing Pearson correlation coefficients. The resulting FC maps underwent fisher’s-z transformation before further statistical analysis.

### Statistical Analysis

To examine the differential SN responses to heat stimulation between CPHP and HC, we performed repeated measure two-way ANOVA with CPHP and HC as a between-group factor and pre- and post-heat stimulation as a within-subject factor using SPM flexi-factorial analysis (Ashburner et al., 2016). We tested the group effect using CPHP > control and control > CPHP contrasts, with height threshold of *p* < 0.01 and cluster threshold of *p* < 0.05 family-wise error (FWE) corrected (Poline et al., 1997).

### Clinical Correlation

To evaluate the relationship between abnormal intrinsic brain connectivity and chronic pain status in CPHP group, we extracted baseline FC between insula and brain regions showing significant group and stimulation interaction effects and correlated with their pre-operative temporal summation scores, pre-operative pain catastrophizing score, CoVAS score during fMRI and 4-month postoperative pain score in CPHP patients. Similarly, we correlated FC changes before and after heat pain stimulations from those regions with the abovementioned clinical scores.

## Results

### Demographic, Clinical Characteristics and Motion Parameters

Demographic and clinical characteristics of the 11 CPHPs and 21 HCs are shown in Table 1. There were no significant differences in age and handedness, but ethnicity composition difference was significant between CPHPs and HCs, with a higher proportion of non-Chinese ethnic individuals in the CPHPs group compared to the HC group (*p* = 0.002). Four-month post-operative pain score difference (*p* < 0.001) and Pre-operative temporal summation score (*p* < 0.011) were also found to be significant between CPHPS and HCs. With regard to the head motions during fMRI scanning, we compared the absolute maximum displacement before and after heat stimulations between CPHPs and HCs using two-way ANOVA. We found no significant between-subject effects between CPHPs and HCs (*F* = 3.424, *p* = 0.074), as well as no significant within-subject effects of between pre and post-heat stimulation (*F* = 0.275, *p* = 0.604), on head motion parameters. Further, a test of interactions between the two factors was not significant (*F* = 0.715, *p* = 0.405; Table 1).

**Table 1:** Demographic and clinical characteristics.

Characteristic	CPSP patients (*n* = 11)	Controls (*n* = 21)	*P*-value
Gender (female/male), *n*	11/0	21/0	N.A.
Race, *n*	6/3/1/1	21/0/0/0	0.002*
(Chinese/Malay/Indian/Others)			
Age (years), mean (SD)	47.6 (6.49)	47.3 (6.28)	0.811
Weight (kg), mean (SD)	65.2 (14.7)	65.5 (9.74)	0.691
Height (cm), mean (SD)	155 (5.19)	159 (4.90)	0.144
Handedness (right/left), *n*	11/0	21/0	N.A.
ASA classification (I/II), *n*	3/8	10/11	0.450
Type of hysterectomy, *n*	5/6	11/10	0.710
(abdominal/laparoscopic)		
Pre-operative pain catastrophizing score (SD)	21.7 (10.92)	14.8 (10.29)	0.059
Pre-operative temporal	16.0 (16.25)	5.29 (13.47)	0.011*
summation score (SD)
CoVAS (SD)	46.5 (31.55)	44.8 (40.27)	0.550
4-month postoperative pain	4.91 (1.64)	0.24 (0.54)	<0.001*
score, mean (SD)
Head motion parameters			
Absolute max displacement			
Pre-stimuli scan (SD)	0.871 (0.580)	0.530 (0.413)	
Post-stimuli scan (SD)	0.731 (0.580)	0.562 (0.359)	

Values are presented as number of subjects or mean (standard deviation). CPSP, chronic postsurgical pain; ASA, American Society of Anesthesiologists; CoVAS, continuous visual analog scale; Mann Whitney U test and Fisher exact tests were used to assess group difference in continuous and categorical variables, respectively. *Represent a significant difference in ethnicity composition (ethnicity comparison based on Chinese vs. non-Chinese), pre-operative mechanical temporal summation score and 4-month postoperative pain score between chronic post-hysterectomy pain (CPHP) subjects and controls (p < 0.05).

### Group Difference in Insular Functional Connectivity Changes Following Heat Pain Stimulation in CPHP Patients and Controls

**Figure 1:**
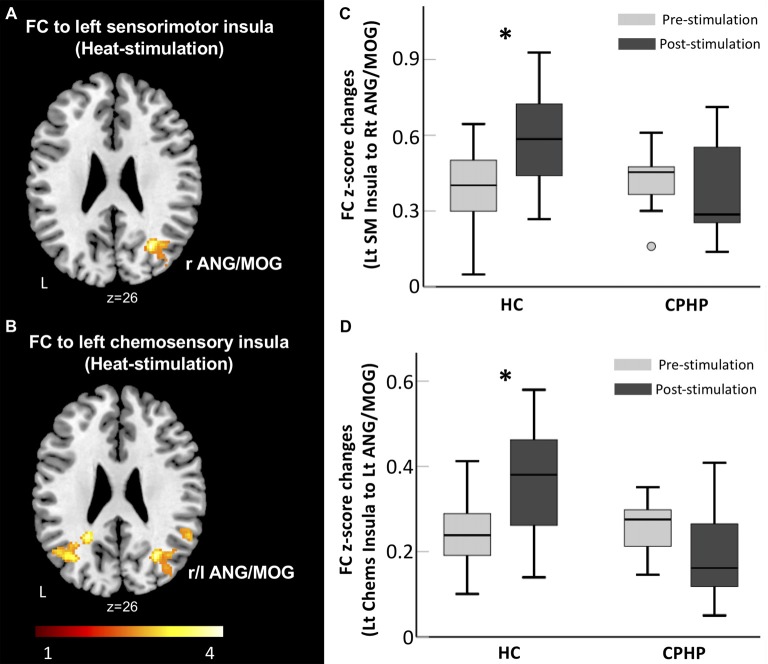
Lack of insular functional connectivity (FC) increase following heat pain stimulation in CPHP. Brain region showing significant interaction effects of Group (HC vs. CPHP) × Stimulation (Pre- vs. Post-heat pain stimulation) in FC (height threshold of *p* < 0.01 and cluster threshold of *p* < 0.05 family-wise error (FWE) corrected) by seeding at the left sensorimotor insula **(A)** and left chemosensory insula **(B)**. Bar plots showing the mean FC between the left sensorimotor insula and right MOG **(C)** and between left chemosensory insula and left AnG **(D**; error bars represents standard error). Abbreviations: HC, healthy control; CPHP, chronic post-hysterectomy pain; MOG, middle occipital gyrus; AnG, angular gyrus; SM, sensorimotor; Chems, chemosensory; r, right; l, left. *Indicates significant difference between pre- and post-stimulation functional connectivity (*p* < 0.05).

We found a significant group and simulation interaction effect across the two groups. In the HC group, FC between left sensorimotor insula (ipsilateral to heat pain stimulus) and right angular and middle occipital gyrus (MOG) increased after heat pain stimulation (Figures 1A,C). FC between the left chemosensory insula and bilateral angular and MOG (Figures 1B,D) increased following heat pain stimulation. In contrast, the CPHP group did not have such a pattern.

### Associations Between Functional Connectivity Changes and Clinical Pain Scores

**Figure 2:**
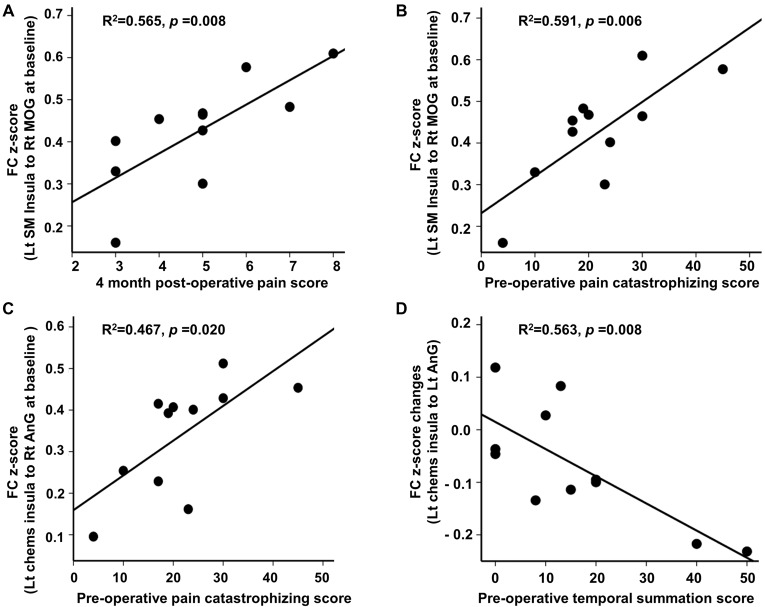
Correlation between insular FC at baseline or changes following heat pain stimulation and clinical scores. Associations were reported at *p* < 0.05 corrected for clinical scores (*n* = 4) for panel **(A,B,D)**, and *p* < 0.05 uncorrected for panel **(C)**. **(A–C)** Post-operative pain score and pre-operative pain catastrophizing score showed positive correlations with insular FC at baseline before the heat pain stimulation in CPHP patients. **(D)** Higher pre-operative temporal summation score correlated with lower increase in insular FC (between left chemosensory insula and left angular gyrus) after heat pain stimulation in CPHP patients. Abbreviations: FC, functional connectivity; SM, sensorimotor; Chems, chemosensory; MOG, middle occipital gyrus; AnG, angular gyrus; Lt, left; Rt, right.

At baseline, we found significant associations between FC and clinical pain-related scores in CPHP patient group, including FC between left sensorimotor insula and right MOG was positively related to 4-month post-operative pain scores (*r*^2^ = 0.565; *p* < 0.05, Figure 2 top left); FC between left sensorimotor insula and right MOG (*r*^2^ = 0.591; *p* < 0.05, Figure 2 top right) and FC between left chemosensory insula and right angular gyrus (*r*^2^ = 0.467; *p* < 0.05, Figure 2 bottom left) were positively associated with pre-operative pain catastrophizing scale score. Following heat pain stimulation, we found that lower increase in insula FC (between the left chemosensory insula and left angular gyrus) after stimulation was associated with higher pre-operative temporal summation scores in CPHP patients (*r*^2^ = 0.563; *p* < 0.05, Figure 2 bottom right). To evaluate the influence of the outlier (referring to Figure 1, panel **C**, box plot of pre-stimulation FC in CPHP), we repeated the correlation analysis after excluding the outlying data point. The results remained significant (panel **A**, *r* = 0.728, *p* = 0.017; panel **B**, *r* = 0.651, *p* = 0.042). No association was found between FC and COVAS score during heat pain stimulation.

## Discussion

In the present study, we examined the insular FC in CPHP using resting state fMRI approach. Following heat pain stimulation, we observed increased insular FC in HC but not in CPHP group. Furthermore, FC abnormalities in CPHP group were correlated with their clinical severity that the smaller FC increment after heat stimulation was related to higher clinical pain-related scores. To our knowledge, this is the first study to look at intrinsic brain connectivity before and after provoked pain stimulation in patients presented with early CPSP. These findings suggest, at the level of large-scale network connectivity, CPHP patients may have lost the normal neural response toward acute pain stimulus, evidenced by the abnormalities in the insular processing after noxious input.

### Reduced Insular Connectivity Changes After Pain Stimulation in Patients With Chronic Post-surgery Pain

We found increased FC between left chemosensory/sensorimotor insula and other cortical regions, including MOG and ANG, following pain stimulation in healthy subjects. The insula is an important cortical structure that is involved in somatosensory, autonomic, interoceptive, salience and cognitive processes (Craig, 2009; Kurth et al., 2010; Borsook et al., 2016). Prior work has highlighted the important role of the insula in the physiological processing and neural response toward pain (Becerra et al., 1999; Peyron et al., 2000; Roberts et al., 2008). Studies have further described the distinct roles of the anatomical divisions of the insula in pain; mid and posterior insula involved in the actual experience of pain and anterior insula involved in anticipation and empathizing with others’ pain (Singer et al., 2009). The increased connectivity between the sensory subfields of the insula and other cortical regions of higher sensory processing following heat pain stimuli in HC group could be a healthy neural response that appropriate neural resources being recruited to manage noxious inputs (Palaniyappan and Liddle, 2012).

Specifically, increased insular-MOG FC supports the involvement of the occipital region in pain processing or modulation. The occipital region has been implicated in the modulation of pain through activation of descending tract of dorsolateral funiculus. Involvement of the occipital cortex in descending inhibitory mechanism was well demonstrated (Reis et al., 2010). In fMRI studies, BOLD responses following thermal stimulation were also observed in occipital regions and believed to reflect the early processing of the noxious stimulus (Atlas et al., 2014).

The angular gyrus is well known to be involved in a multitude of cognitive functions including attention, language and semantic memory (Binder et al., 2009; Uddin et al., 2010). It is also the functional hub of the DMN which is involved in monitoring the internal environment for salient events (Raichle et al., 2001; Raichle and Snyder, 2007). DMN suppression during cognitive tasks is a common observation in healthy individuals. Decreased suppression or hyperactivity of DMN has been observed in multiple pain conditions on tasks (Seminowicz et al., 2011; Weissman-Fogel et al., 2011; Mathur et al., 2015) or at rest (Baliki et al., 2008; Ceko et al., 2015b). In addition to the early works, our findings showed the failure of SN to mobilize the DMN following heat pain stimulation in CPSP, suggesting a functional re-organization between the two large-scale neuronal networks in the chronic pain condition (Kucyi et al., 2014; Ceko et al., 2015a).

In our study, the increased SN connectivity following pain stimulation was not observed in CPHP, suggesting that the normal pain-induced neural response may be blunted or abolished in CPHP. Our findings support the idea that in CPHP, normal sensory processing pathway was dysregulated, result in altered brain functional circuit secondary to neuroplasticity. This is consistent with previous studies in which atypical insular responses have been demonstrated in several neuropsychiatric conditions, including schizophrenia (Palaniyappan and Liddle, 2012; Moran et al., 2013) and Alzheimer’s disease (Zhou et al., 2010). A possible explanation for these findings is that chronic pain stimulus has caused functional saturation or over-sensitization of pain processing pathway that prevents further FC increment upon heat pain stimulation. In other words, CPHPs may become so accustomed to continuous or ongoing post-surgical pain that the brief pain stimulus evoked in the experiment failed to produce further FC increment as compared to HC who were not accustomed to such pain.

### Aberrant Insular Connectivity Associated With Worse Behavioral Outcomes

Our study showed that in CPHP, the extent of FC increases toward provoked pain stimulus negatively correlated with higher pre-operative temporal summation score. This is consistent with the above findings that CPHP patients showed blunted FC response toward provoked pain. Temporal summation score is a dynamic test for central sensitization (Loeser and Treede, 2008) and diffuse noxious inhibitory control (DNIC; Yarnitsky et al., 2008). Higher mechanical temporal summation has been shown to predict acute provoked pain after thoracotomy, suggesting central pain augmentation or sensitization (Herrero et al., 2000; Landau et al., 2010). These correlation results not only supported the validity of mechanical temporal summation to predict chronic pain development but also to affirm the underlying relationship between pain-evoked altered insular FC changes and physical pain testing. Within CPHP, we also found pre-stimuli FC strength was positively correlated to pre-operative pain catastrophizing and to 4-month post-operative clinical pain scores. This supports the aforementioned hypothesis that the pain preprocessing pathways at the cortical level in CPSP patients are over-sensitized at baseline due to chronic persistent pain stimuli.

By identifying and quantifying insular dysfunctionality in the early stage of CPSP development, we can have a better understanding about neural mechanism of pain, and with the invention of effective therapeutic measures, we may possibly halt the progression of early to chronic CPSP. Further, insular FC changes and dysfunctionality can also potentially be used as a surrogate measure of pain score, thereby allow more objective monitoring of the therapeutic response of CPSP toward analgesia or other treatment.

### Limitations and Future Work

The present study had some limitations. Although only substance free participants were recruited, the study did not control for several comorbidities commonly associated with chronic pain condition such as depression and anxiety, which may potentially confound our findings. In view of the relatively small sample size, to reduce multiple comparisons the current study only focused on insular FC using a seed-based approach, which may not be able to provide a complete picture of whole-brain functional network changes. A more data-driven approach, such as whole brain FC analysis, would allow us to examine the involvements of other neural pathways in chronic pain modulations. Similarly, because of the small sample size, the findings from correlation analysis should be regarded as exploratory. Further work with large sample size is needed to address these issues.

In conclusion, our findings highlight the insular FC abnormalities before and after heat pain stimulation in CPHP patients underlying their clinical symptoms. Future studies are needed to help stratify patients according to comorbidities and pain levels to characterize specific altered brain functional connectome underlying CPSP and develop such method to help intervention development.

## Author Contributions

All authors listed have made a substantial, direct and intellectual contribution to the work, and approved it for publication.

## Conflict of Interest Statement

The authors declare that the research was conducted in the absence of any commercial or financial relationships that could be construed as a potential conflict of interest.
